# Analysis of a large single institution cohort of related donors fails to detect a relation between SDF1/CXCR4 or VCAM/VLA4 genetic polymorphisms and the level of hematopoietic progenitor cell mobilization in response to G-CSF

**DOI:** 10.1371/journal.pone.0228878

**Published:** 2020-03-05

**Authors:** Sylvain Garciaz, Patrick Sfumato, Angela Granata, Anne-Marie Imbert, Claire Fournel, Boris Calmels, Claude Lemarie, Jacques Chiaroni, Didier Blaise, Jean-Marie Boher, Christophe Picard, Christian Chabannon, Julie di Cristofaro

**Affiliations:** 1 Institut Paoli-Calmettes, Comprehensive Cancer Center, Marseille, France; 2 Aix-Marseille Univ, Inserm, CNRS, Institut Paoli-Calmettes, CRCM, Marseille, France; 3 Centre d’Investigations Cliniques de Marseille, module Biothérapies, Inserm CBT, Marseille, France; 4 Etablissement Français du Sang PACA Corse, Biologie des Groupes Sanguins, Marseille, France; 5 Aix Marseille Univ, CNRS, EFS, ADES, Marseille, France; Goethe University Frankfurt, GERMANY

## Abstract

We studied a cohort of 367 healthy related donors who volunteered to donate their hematopoietic stem cells for allogeneic transplantation. All donors were homogeneously cared for at a single institution, and received rhG-CSF as a mobilization treatment prior to undergoing apheresis. Peripheral blood CD34+ cell counts were used as the main surrogate marker for rhG-CSF induced mobilization. We searched whether inter-individual variations in known genetic polymorphisms located in genes whose products are functionally important for mobilization, could affect the extent of CD34+ mobilization, either individually or in combination. We found little or no influence of individual SNPs or haplotypes for the SDF1, CXCR4, VCAM and VLA4 genes, whether using CD34+ cell counts as a continuous or a categorical variable. Simple clinical characteristics describing donors such as body mass index, age and possibly sex are more potent predictors of stem cell mobilization. The size of our cohort remains relatively small for genetic analyses, however compares favorably with cohorts analyzed in previously published reports suggesting associations of genetic traits to response to rhG-CSF; notwithstanding this limitation, our data do not support the use of genetic analyses when the choice exists of several potential donors for a given patient.

## Introduction

Allogeneic stem-cell transplantation (ASCT) is a curative treatment for patients with severe hematological malignancies. Several sources of stem cells can be used, including bone marrow (BM), peripheral blood (PB) and umbilical cord blood. PB cell collection presents several advantages: leukapheresis is a moderately invasive and semi-automated procedure that can be performed on an outpatient basis and does not require access to the operating room nor general anesthesia; infusion of PB cells to the recipient is associated with rapid engraftment and hospital discharge, both after myeloablative and reduced intensity conditioning regimen. [1, 2] Although, the relationship between the numbers of infused CD34+ cells and recipient engraftment and outcome remains controversial [3, 4], most transplant programs expect that the collected cell graft will contain at least a defined minimal number of progenitors, and some will possibly cap the number of infused CD34+ cells. These objectives can be reached only when donors CD34+ cells are appropriately mobilized through the administration of granulocyte colony-stimulating factor (G-CSF). The number of CD34+ cells mobilized into PB varies significantly among donors, mainly depending on clinical factors as age, gender or weight. [5, 6] Genetic susceptibility may also influence the quality of mobilization. Indeed, several BM proteins are involved in stem-cell homing and G-CSF-induced mobilization, including CXCR4 and its ligand SDF1 (CXCL12) [7, 8] and VCAM1 and its ligand VLA4. [9, 10] Interactions of these two receptor-ligand pairs are disrupted following G-CSF treatment, and structural or functional variations in these molecules may influence response to G-CSF. [11–13] Some of these inter-individual variations may be appreciated through single nucleotide polymorphisms (SNPs). In contexts other than HSCT, many studies have evidenced a relation between SNPs and the variability in response to drug administration. [14, 15] There are limited evidences suggesting that similar patterns may exist for CD34+ cell mobilization in response to rhG-CSF, either for patients or healthy donors who are preparing to undergo apheresis prior to autologous or allogeneic hematopoietic cell transplantation respectively. [11–13] We conducted a retrospective analysis of the association between *SDF1/CXCR4* and *VCAM/VLA4* genetic polymorphisms and CD34+ cells mobilization in healthy related donors.

## Patients & methods

### Donor selection and care

The study includes three hundred and sixty-seven adult healthy donors who donated their blood mononuclear cells to related patients who received allogeneic transplantation between 1997 and 2016 and were cared for at a single institution: Institut Paoli-Calmettes, the comprehensive cancer center in Marseille (see Table 1). All donors were identified, screened and collected in accordance with national or international regulations, institutional policies, EFI and EBMT/FACT-JACIE prescriptions, including transparent information on HLA typing, donation and their rights to consent. The project was approved by the "Comité d'Orientation Stratégique" (COS; Internal Review Board) at the Direction de la Recherche Clinique et de l'Innovation (DRCI), Institut Paoli-Calmettes. All patients and donors involved in the adult transplantation program at Institut Paoli-Calmettes provided informed written consent for the use of their personal data as per EBMT and FACT-JACIE requirements, in compliance with national and European regulations.

**Table 1:** Donors clinical and biological data.

*Variable*	*Classes*	*Statistics*	*All (n = 367)*
Gender	Male	n (%)	209 (56.95)
	Female	n (%)	158 (43.05)
Age		n	365
		Mean (SD)	50.45 (12.29)
		Median [Min—Max]	52.00 [18.00–78.00]
		Number of missing data	2
Height		n	329
		Mean (SD)	169.9 (9.118)
		Median [Min—Max]	170.0 [150.0–195.0]
		Number of missing data	38
Weight		n	334
		Mean (SD)	73.87 (15.44)
		Median [Min—Max]	72.00 [44.00–130.0]
		Number of missing data	33
Body Mass Index		n	329
		Mean (SD)	25.50 (4.620)
		Median [Min—Max]	25.00 [16.00–45.00]
		Number of missing data	38
G-CSF		n	367
		Mean (SD)	674.1 (132.9)
		Median [Min—Max]	600.0 [480.0–960.0]
G-CSF/kg		n	334
		Mean (SD)	9.441 (1.849)
		Median [Min—Max]	9.375 [4.615–15.00]
		Number of missing data	33
PB CD34+ cells /microL at day 5		n	367
		Mean (SD)	67.36 (41.73)
		Median [Min—Max]	59.20 [4.000–237.6]

In preparation for apheresis, donors received daily SQ injections of rhG-CSF in the evening as per institutional procedures. Prior to September 2009, all donors received a 600 μg daily dose independently of their weight. Starting in September 2009, the G-CSF daily dose was adjusted to donor’s weight to approximately match the 10μg/kg/day dosing, as recommended per rhG-CSF label (daily doses were 480, 600, 780 or 900μg). Counseling was provided on expected side effects, particularly bone pain, and prophylactic paracetamol prescribed to reduce pain. Circulating CD34+ cells were first counted at day 5, after 4 evening injections of rhG-CSF, using a single-platform flow-cytometry based technique as previously described. [4]

No donor infectious or inflammatory manifestations were reported in the 8 days preceding donation. All CRP were negative (<6 mg/l).

### VCAM rs1041163, VLA4 rs1449263 and CXCR4-rs2680880 genotyping

Genomic DNA (gDNA) was extracted from a 200-μl total blood sample using the QIAmp Blood DNA Mini kit (Qiagen, Courtaboeuf, France) according to manufacturer’s instructions.

*VCAM1*-rs1041163 (T>C), *VLA4*-rs1449263 (A>G) and *CXCR4*-rs2680880 (A>T) were analyzed by direct sequencing after PCR amplification. PCR amplification was performed on 50 ng of gDNA in a final volume of 25 μL containing 1x PCR buffer, 1.5 mM MgCl2, 0.2 mM of dNTPs, 0.1 unit of Taq DNA-polymerase (Invitrogen, France) and 0.16 μM of each primer. Primers designed using the Primer 3 program (http://bioinfo.ut.ee/primer3-0.4.0/primer3/). The *VCAM*, *VLA4* and *CXCR4* PCR primer sequences were respectively 5’ATTGGCCATTGTCTTTGAGC3’ and 5’GATGCTGTTCTAGGGTGTGG3’; 5’TGCCCACTATATGCCAAAAA3’ and 5’AGGGAGCCATCAGAGGAAAC3’; and 5’GGAAAAGATGGGGAGGAGAG3’ and 5’CACTTCCAATTCAGCAAGCA3’. Amplification was carried out as follows: 1 cycle at 95°C for 5 min; 30 cycles at 95°C for 30 sec, Tm (57°C for *VCAM* and *VLA4* and 53°C for *CXCR4*) for 30 sec, and 72°C for 1 min; and 1 cycle at 72°C for 7 min. PCR amplification was checked by agarose electrophoresis and PCR fragments (length *VLA4*: 250 bp; *VCAM*: 227 bp; *CXCR4*: 159 bp) were sequenced using a Big Dye Terminator V1.1 kit (Invitrogen) with each PCR primer according to the manufacturer’s protocol. Sequences were analyzed using the Codon Code Aligner program (Codon Code Corporation, Massachusetts) as previously described. [16–17]

### CXCR4 (rs12691874, rs16832740, rs2228014) and SDF1 (rs1413519, rs1801157, rs2297630, rs266085, rs266087) genotyping

A homemade multiplex primer extension method was used to simultaneously analyze 8 SNPs of the *CXCR4* and *SDF1* genes (*CXCR4*-rs12691874 A>G, *CXCR4*-rs16832740 T>C, *CXCR4*-rs2228014 C>T, *SDF1*-rs1413519 G>C, *SDF1*-rs1801157 G>A, *SDF1*-rs2297630 G>A, *SDF1*-rs266085 C>T, *SDF1*-rs266087 G>A). All primers were designed using the Primer 3 program. Multiplex PCR using primers flanking the target SNPs were performed on 100 ng of genomic DNA in a final volume of 25 μL containing PCR Qiagen Master Mix (Qiagen, France) and 0.8M of each primer (Table 2). Amplification was carried out as follows: 1 cycle at 95°C for 15 min; 30 cycles at 95°C for 30 sec, 57°C for 45 sec, and 72°C for 60 sec; and 1 cycle at 72°C for 10 min. After control on agarose gel, 5 μl of PCR product was incubated with 0.5 units of thermosensitive alkaline phosphatase and 1 unit of exonuclease-I (Euromedex; France) for 15 min at 37°C followed by 15 min at 80°C to remove unincorporated primers and dNTPs. The second step was a multiplex extension reaction performed using the SNapShot kit (Invitrogen) according to manufacturer’s protocol in a final volume of 10 μL containing 3 μL of the PCR product, 5 μL of SNapShot mix, and extension primers (Table 3). The reaction program was 25 cycles at 95°C for 10 seconds, 50°C for 5 seconds, 60°C for 30 seconds. Snap Shot extension primer data were analyzed using GeneMapper v4.0 with specific detection parameters as previously described. [17]

**Table 2:** PCR primers sequence and concentration used to co-amplify 6 PCR fragments encompassing 8 SNPs of the 8 SNPs of the *CXCR4* and *SDF1* genes.

SNP	Primer sequence, Forward (F) and Reverse (R)		Product Size (pb)
*SDF1* rs2297630	F	GGGCAGCCTTTCTCTTCTTC	193
	R	CCTGAGACTGAAGGCACAGTT	
*SDF1* rs266087	F	ACTGCAAGTGTGTGGAGCTG	248
	R	CCACACCGGCTTCTGTATTT	
*SDF1* rs266085	F	CTTTAGCTTCGGGTCAATGC	246
	R	GCAATGGAACTTCCTGCACT	
*SDF1* rs1801157	F	CTGGGCAAAGCCTAGTGAAG	209
	R	AGAACGTGGAGGATGTGGAG	
*SDF1* rs1413519	F	CTGTCCCAGAAGCCTGAAAG	248
	R	TAGCGCCATGTGCTTCTAAA	
*CXCR4* -rs16832740-	F	CTCGCCTGAAAATGGAGCTA	305
	R	CTTCTTCACATTGGGGATCC	
*CXCR4* -rs2228014	F	CCGTGGCAAACTGGTACTTT	188
	R	GACGCCAACATAGACCACCT	
*CXCR4-*12691874	F	TTGGTGGTGACCTCAGACAG	208
	R	CCCACCCTGTTCTTTTGCTA	

**Table 3:** Extension primers sequence and concentration used to genotype *CXCR4*-rs12691874 A>G, *CXCR4*-rs16832740 T>C, *CXCR4*-rs2228014 C>T, *SDF1*-rs1413519 G>C, *SDF*1-rs1801157 G>A, *SDF1*-rs2297630 G>A, *SDF1*-rs266085 C>T, *SDF1*-rs266087 G>A.

SNP	Primer sequence, Forward (F) and Reverse (R)		Primer Size (b)	Final Concentration (μM)
*SDF1* rs1413519	F	AGGCCCCAGAACAGAAGCTA	20	2.44
*SDF1* rs2297630	F	4T-GTTCGTCTCAGTCTGCATAA	24	0.39
*CXCR4* rs16832740	F	12T-GCCTGGAATTTCAATATACA	32	1.95
*SDF1* rs266087	F	16T-TAAGAGAGGAAGTGGAGGGC	36	2.44
*SDF1* rs266085	F	24T-TGCATCCGCTCCCCCAACAC	44	2.44
*SDF1* rs1801157	F	32T-TCTCCATCCACATGGGAGCC	52	0.78
*CXCR4* rs2228014	F	20T-CTGGACCGCTACCTGGCCAT	40	1.95
*CXCR4* 12691874	R	28T-ACAGTCCACAGGGCTCTAGG	48	1.95

### Statistical analyses

Donor’s associated data—categorized into biological and clinical data including age, height, weight, IMC, sex, G-CSF total dose, G-CSF dose/kg and peripheral blood CD34+ cell counts—are described in Table 1.

#### Allelic frequencies and haplotype estimation

Missing data at a locus led to the exclusion of the concerned sample from further analyses at the given locus. No multiple imputations were used. Allelic and two or more loci haplotype frequencies were estimated using an EM algorithm implemented in the Gene[Rate] computer tools. [12] Deviations from Hardy-Weinberg equilibrium (HWE) were tested using a nested likelihood model. [13]

Haplotypes frequencies based on genotype of each SNPs of a same gene, i.e. *SDF1* and *CXCR4*, were estimated by Gene[rate] computer tool package with no a priori. For allelic and two or more loci frequency estimations, all putative homozygotes were considered either true homozygotes or heterozygotes for the observed allele, and an undefined or undetectable (‘blank’) allele as previously described. [16]

Based on this haplotype estimation, main haplotypes, with a cumulated frequency higher than 98%, were *a priori* encoded for each gene and genotype data were reanalyzed according to this new nomenclature. Using an in-house computer program, data output files (.txt) were formatted into files readable by the “Phenotype” application of the Gene[rate] computer tool package.

#### Biological parameters statistical testing

Statistical analyses were performed using SPSS software (SPSS 19.0 for Windows; SPSS Inc., Chicago, IL) and the R software version 3.0.3 associated to random forest SRC package [18]. The primary endpoint was influence of SNPs on peripheral blood CD34+ cell count/mL on day 5 of G-CSF treatment; CD34+ cell counts were considered as continuous and categorical variables in separate analyses. For continuous variables, median and extreme values are presented. Differences in medians have been analyzed with the t-test for comparisons of two independent samples in univariate analyses or with one-way anova for multiple comparisons. t-tests were considered as significant when two-tailed p-values were < 0.05, except for clinical or molecular factors that has already been associated with a modification with mobilization in previous studies (age, gender, BMI and *VCAM1*-rs1041163 CC homozygous variant). [14] In these case, given the expected influence on mobilization, we used a one-tailed p-value < .05. No adjustment for multiple tests was performed.

#### Linear regression

Two random forests for linear regression [19] were used to evaluate importance of clinical and molecular variables on a higher CD34+ cells count. The first model considered SNP classification as molecular variables and the second used haplotypes classification. The measure of the prediction accuracy of the Random Forest models was given by the mean squared error (MSE); variables importance (VIMP) was determined using permutation importance measure for Random forest, based on out-of-bag (OOB) estimate of prediction error: for a given variable, OOB cases (the original data left out from the bootstrap sample used to grow the tree; approximately 1/3 of the original sample) are randomly permuted in this variable and the prediction error is recorded. The VIMP of this variable is defined as the difference between the perturbed and unperturbed error rate averaged over all trees. The larger value this difference, the more predictive the variable.

## Results

### Influence of clinical characteristics on CD34+ cell mobilization

**Fig 1:**
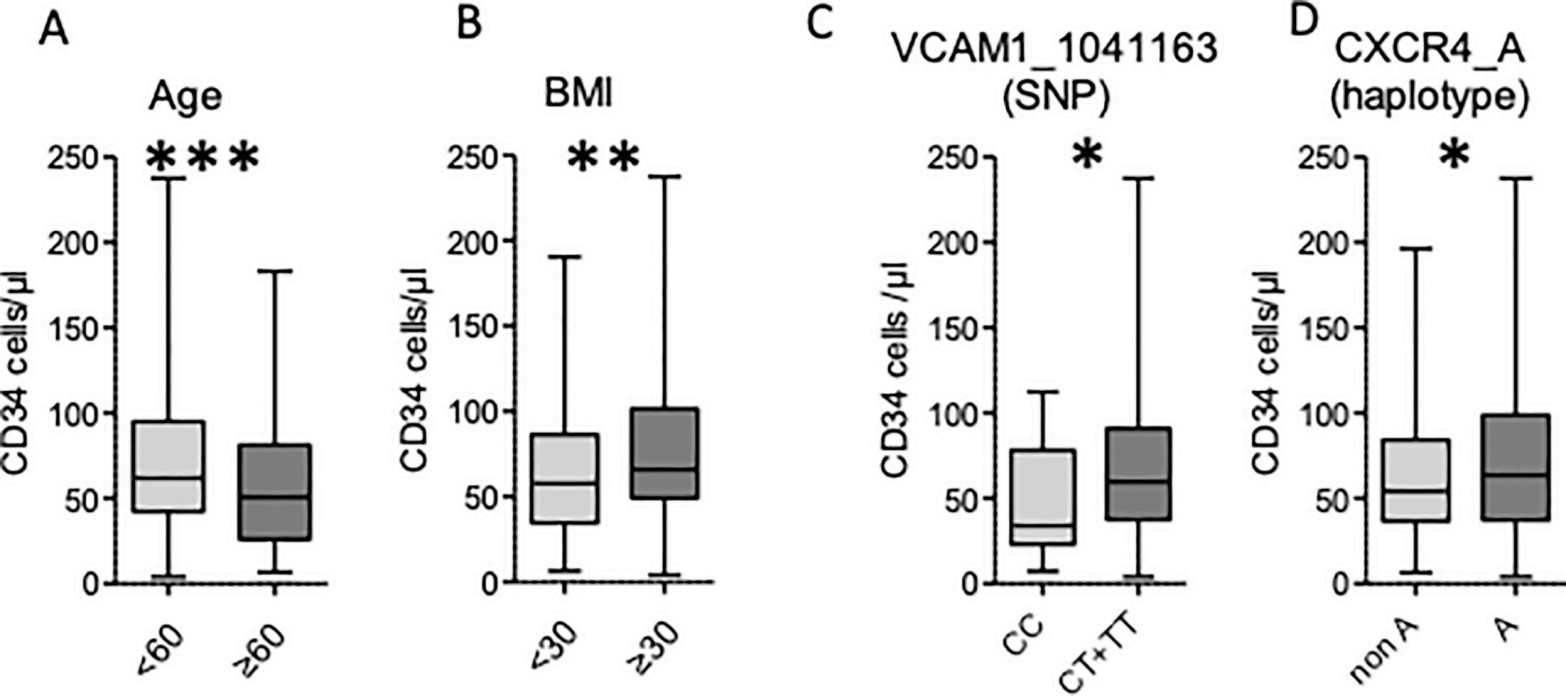
Clinical and molecular factors influencing CD34+ mobilization in univariable analyses.

Among the 367 donors, 158 were females and 209 males. Median age and BMI were 51 (18–77) and 25 (12–44), respectively. The mean number of CD34+ cells/μl after four injections of rhG-CSF was 59.2 [4–237.6]. We first carried out a univariate analysis to study the impact of gender, age and body mass index (BMI) on CD34+ cell mobilization (Table 4). An age younger than 60 (n = 269) and a BMI ≥30 (n = 54) were both associated with a higher CD34^+^ cell count (60.9 [4–237.6] *vs* 51.4 [6.7–183.3], p = .001 and 67.4 [4–237.6] *vs* 57.2 [6.5–190.6], p = .005, respectively, Fig 1A and 1B). Men showed a trend to a better mobilization in comparison with women, although the difference was not significant (62 [7.1–237.6] *vs* 54.5 [4–193.9], p = 0.08).

**Table 4:** Relation between donor clinical characteristics and observed CD34+ cell mobilization after 4 injections of rhG-CSF.

Clinical characteristics		Median CD34+ cells/μl	range	Two-tailed p value	One-tailed p value
gender	female (n = 158)	54.5	4–193.9	0.16	0.08
	male (n = 209)	62	7.1–237.6		
age (years)	<60 (n = 269)	60.9	4–237.6	0.002	0.001
	≥60 (n = 76)	51.4	6.7–183.3		
BMI	<30 (n = 232)	57.2	6.5–190.6	0.01	0.005
	≥30(n = 54)	67.4	4–237.6		

### Impact of VCAM/VLA4 and SDF1/CXCR4 SNPs on CD34 positive cell mobilization

We next searched for a relation between each genetic SNP and PB CD34+ cell counts was analyzed in univariate analyses. Allelic frequencies are described in Table 5. We did not observe deviations from the Hardy-Weinberg distribution. The CT or TT variants for the *VCAM1*-rs1041163 SNP (n = 347) were associated with a higher CD34+ cell count than the homozygous CC variant (59.6 [4–237.6] *vs* 34 [7.4–112.3], p = 0.03) (Fig 1C and Table 6). We did not find any other significant association between SNPs and mobilization (Table 6). We also used the CD34 count as a categorical variable taking 30 CD34+ cells/μl as a cut-off, assuming it is clinically relevant in pinpointing the poor mobilizer (<30 CD34+ cells/μl). This analysis failed to find a significant association between VCAM/VLA4 or SDF1/CXCR4 polymorphism and mobilization (Table 7).

**Table 5:** Allele frequency for *VCAM*, *VLA4*, *CXCR4* and *SDF1* in the donor cohort.

*SNP*	*Allele*	*n*	*FQ*
*VCAM*_rs1041163	CC	19	0.052
	CT	102	0.278
	TT	245	0.669
*VLA4*_rs1449263	GG	69	0.188
	GA	188	0.513
	AA	109	0.298
*CXCR4*_rs2680880	AA	35	0.095
	AT	157	0.428
	TT	175	0.478
*CXCR4*_rs12691874	AA	74	0.202
	AG	184	0.502
	GG	108	0.295
*CXCR4*_rs16832740	CC	10	0.273
	CT	101	0.276
	TT	255	0.696
*CXCR4*_rs2228014	CC	330	0.904
	CT	35	0.096
	TT	0	0
*SDF1*_rs1413519	CC	15	0.409
	GC	109	0.298
	GG	242	0.661
*SDF1*_rs1801157	AA	22	0.061
	GA	116	0.317
	GG	228	0.622
*SDF1*_rs2297630	AA	19	0.052
	GA	108	0.295
	GG	239	0.653
*SDF1*_rs266085	TT	71	0.193
	CT	170	0.464
	CC	125	0.342
*SDF1*_rs266087	AA	70	0.191
	GA	170	0.464
	GG	126	0.344

**Table 6:** Impact of *VCAM/VLA4* and *SDF1/CXCR4* SNP on CD34+ cell mobilization using CD34+ as a continuous variable.

**SNP**		median	range	two-tailed p value
*VCAM*_rs1041163	CC (n = 19)	34	7.4–112.3	-
	CT (n = 102)	58,6	6.5–237.6	0,16
	TT (n = 245)	60,1	4–193.9	0,04
	CT+TT (n = 347)	59,6	4–237.6	0,06
*VLA4*_rs1449263	GG (n = 69)	53,9	7.4–103.4	-
	GA (n = 188)	59,9	4–237.6	0,33
	AA (n = 109)	59	6.7–186.8	0,59
	GA+AA (n = 297)	59	6.7–186.8	0,39
**SNP**		median	range	P
*CXCR4*_rs2680880	AA (n = 35)	53,8	21.7–162	-
	AT (n = 157)	59,6	6.5–186.8	0,65
	TT (n = 175)	59,6	4–237.6	0,25
	AT+TT (n = 332)	59,6	4–237.6	0,31
*CXCR4*_rs12691874	AA (n = 74)	57,1	6.8–193.3	-
	AG (n = 184)	58,9	6.5–237.6	0,99
	GG (n = 108)	60,6	4–190.6	0,78
	AG+GG (n = 292)	59,5	4–237.6	0,91
*CXCR4*_rs16832740	CC (n = 10)	60,6	32.2–120.9	-
	CT (= 101)	59,2	6.5–186.8	0,71
	TT (n = 255)	59	4–237.6	0,71
	CT+TT (n = 356)	59,15	4–237.6	0,79
*CXCR4*_rs2228014	CC (n = 330)	59,6	4–237.6	-
	CT (n = 35)	53,8	7.1–175.8	0,49
**SNP**		median	range	P
*SDF1*_rs1413519	CC (n = 15)	56,4	4–107.5	
	GC (n = 109)	59,6	6.8–237.6	0,22
	GG (n = 242)	59,5	6.5–196.3	0,15
	GC+GG (n = 351)	59,6	6.5–196.3	0,15
*SDF1*_rs1801157	AA (n = 22)	52,35	12.7–177.4	
	GA (n = 116)	59,2	7.1–196.3	0,4
	GG (n = 228)	59,6	4–237.6	0,47
	GA+GG (n = 344)	59,6	4–237.6	0,44
*SDF1*_rs2297630	AA (n = 19)	49,4	8.9–119.3	
	GA (n = 108	59,4	6.5–190.6	0,36
	GG (n = 239)	59,4	4–237.6	0,31
	GA+GG (n = 347	59,4	4–237.6	0,28
*SDF1*_rs266085	TT (n = 71)	64,8	6.7–193.9	
	CT (n = 170)	59,6	8.7–237.6	0,72
	CC (n = 125)	57,8	4–183.9	0,4
	CT+CC (n = 295)	59,1	4–237.6	0,67
*SDF1*_rs266087	AA (n = 70)	63,2	6.7–193.9	
	GA (n = 170	59,6	8.7–237.6	0,77
	GG (n = 126)	58,4	4–183.9	0,46
	GA+GG (n = 296)	59,15	4–183.9	0,73

**Table 7:** Impact of *VCAM/VLA4* and *SDF1/CXCR4* SNP on CD34+ cell mobilization using CD34+ as a categorical variable.

*Test*	*Classes*	*Statistics*	*All (n = 367)*	CD34+<*30 (n = 66)*	CD34+≥*30 (n = 301)*	*p-value (wilcoxon or khi^2^)*
Gender	Male	n (%)	209 (56.95)	35 (53.03)	174 (57.81)	0.4778
	Female	n (%)	158 (43.05)	31 (46.97)	127 (42.19)	
Age		n	365	65	300	0.012
		Mean (SD)	50.45 (12.29)	54.23 (14.70)	49.63 (11.57)	
		Median [Min—Max]	52.00 [18.00–78.00]	55.00 [21.00–78.00]	51.00 [18.00–77.00]	
	< = 60	n (%)	291 (79.73)	43 (66.15)	248 (82.67)	0.0027
	>60	n (%)	74 (20.27)	22 (33.85)	52 (17.33)	
		Number of missing data	2	1	1	
BMI		n	329	57	272	0.260
		Mean (SD)	25.50 (4.620)	24.82 (4.268)	25.64 (4.685)	
		Median [Min—Max]	25.00 [16.00–45.00]	25.00 [18.00–45.00]	25.00 [16.00–44.00]	
	< = 25	n (%)	179 (54.41)	33 (57.89)	146 (53.68)	0.5610
	>25	n (%)	150 (45.59)	24 (42.11)	126 (46.32)	
		Number of missing data	38	9	29	
VLA4_rs1449263	A,A	n (%)	109 (29.7)	26 (39.4)	163 (54.1)	0.0867
	G,A	n (%)	188 (51.2)	25 (37.8)	83 (27.6)	
	G,G	n (%)	69 (18.8)	15 (22.7)	54 (17.9)	
		Number of missing data	1		1	
*VCAM*_rs1041163	T,T	n (%)	245 (66.8)	42 (63.6)	203 (67.4)	0.2865
	C,T	n (%)	102 (27.8)	18 (27.3)	84 (27.9)	
	C,C	n (%)	19 (5.2)	6 (9.1)	13 (4.3)	
		Number of missing data	1		1	
*SDF1*_rs1413519	G,G	n (%)	242 (66.12)	45 (68.18)	197 (65.67)	0.8776
	G,C	n (%)	109 (29.78)	18 (27.27)	91 (30.33)	
	C,C	n (%)	15 (4.098)	3 (4.545)	12 (4.000)	
		Number of missing data	1		1	
*SDF1*_rs1801157	G,G	n (%)	228 (62.30)	38 (57.58)	190 (63.33)	0.4433
	G,A	n (%)	116 (31.69)	22 (33.33)	94 (31.33)	
	A,A	n (%)	22 (6.011)	6 (9.091)	16 (5.333)	
		Number of missing data	1		1	
*SDF1*_rs2297630	G,G	n (%)	238 (65.21)	43 (65.15)	195 (65.22)	0.9367
	G,A	n (%)	108 (29.59)	19 (28.79)	89 (29.77)	
	A,A	n (%)	19 (5.205)	4 (6.061)	15 (5.017)	
		Number of missing data	2		2	
*SDF1*_rs266085	C,C	n (%)	125 (34.15)	19 (28.79)	106 (35.33)	0.5964
	C,T	n (%)	170 (46.45)	33 (50.00)	137 (45.67)	
	T,T	n (%)	71 (19.40)	14 (21.21)	57 (19.00)	
		Number of missing data	1		1	
*CXCR4*_rs2680880	T,T	n (%)	175 (47.68)	32 (48.48)	143 (47.51)	0.2947
	A,T	n (%)	157 (42.78)	31 (46.97)	126 (41.86)	
	A,A	n (%)	35 (9.537)	3 (4.545)	32 (10.63)	
		Number of missing data	0			
*CXCR4*_rs12691874	G,G	n (%)	108 (29.51)	19 (28.79)	89 (29.67)	0.6591
	G,A	n (%)	184 (50.27)	31 (46.97)	153 (51.00)	
	A,A	n (%)	74 (20.22)	16 (24.24)	58 (19.33)	
		Number of missing data	1		1	
*CXCR4*_rs16832740	T,T	n (%)	255 (69.67)	47 (71.21)	208 (69.33)	0.3216
	C,T	n (%)	101 (27.60)	19 (28.79)	82 (27.33)	
	C,C	n (%)	10 (2.732)		10 (3.333)	
		Number of missing data	1		1	
*CXCR4*_rs2228014	C,C	n (%)	330 (90.16)	60 (90.91)	270 (90.00)	0.8853
	C,T	n (%)	35 (9.563)	6 (9.091)	29 (9.667)	
	T,T	n (%)	1 (0.273)		1 (0.333)	
		Number of missing data	1		1	

### Impact of VCAM/VLA4 and SDF1/CXCR4 haplotypes on CD34+ cell mobilization

#### Allelic frequencies and haplotype estimation

Haplotypes were also investigated to identify better mobilizers. A haplotype is a group of gene variants that are inherited together from a single parent. Haplotypic frequencies based on genotype of each SNPs of a same gene, i.e. *SDF1* and *CXCR4*, estimated by Gene[rate] without *a priori*, are described in Tables 8 and 9. Nine and 8 haplotypes were respectively estimated for SDF1 and CXCR4, and for both genes, 5 haplotypes displayed a cumulated frequency of 99.3% and 98.5% respectively, encoded *SDF1*-A to *SDF1*-E and *CXCR4*-A to *CXCR4*-E. Genotype data reanalyzed according to this *a priori* nomenclature are described in Tables 10 and 11. With this new coding, blank haplotype represented respectively 5.8% and 6.1% for *SDF1* and *CXCR4*.

**Table 8:** *SDF1* haplotypes and their frequencies (FQ) estimated by Gene[rate] based on rs1801157, rs266087, rs2297630, rs266085, rs1413519 observed polymorphisms.

HAPLOTYPE	FQ
**A~A~G~T~G**	0.2158
**G~A~G~T~G**	0.2046
**G~G~A~C~G**	0.1995
**G~G~G~C~C**	0.1868
**G~G~G~C~G**	0.1862
**A~G~G~T~G**	0.0027
**G~A~G~T~C**	0.0017
**G~A~G~C~G**	0.0014
**G~G~G~T~C**	0.0014

**Table 9:** *CXCR4* haplotypes and their frequencies (FQ) estimated by Gene[rate] based on rs16832740, rs2228014, rs2680880 and rs12691874 observed polymorphisms.

HAPLOTYPE	FQ
**T~C~T~G**	0.3263
**T~C~T~A**	0.3103
**C~C~A~G**	0.1587
**T~C~A~A**	0.1414
**T~T~T~G**	0.0486
**T~C~A~G**	0.0062
**C~C~T~G**	0.0047
**C~C~A~A**	0.0019

**Table 10:** Main *SDF1* haplotypes frequencies (FQ) estimated with an a priori by Gene[rate].

HAPLOTYPE	RS1801157	RS266087	RS2297630	RS266085	RS1413519	FQ
*SDF1A*	A	A	G	T	G	0.2026
*SDF1B*	G	A	G	T	G	0.1943
*SDF1C*	G	G	A	C	G	0.1881
*SDF1D*	G	G	G	C	G	0.1782
*SDF1E*	G	G	G	C	C	0.1782
**BLANK**						0.0585

**Table 11:** Main *CXCR4* haplotypes frequencies (FQ) estimated with an a priori by Gene[rate].

HAPLOTYPE	RS16832740	RS2228014	RS2680880	RS12691874	FQ
**CXCR4A**	T	C	T	G	0.3074
**CXCR4B**	T	C	T	A	0.2933
**CXCR4C**	C	C	A	G	0.1524
**CXCR4D**	T	C	A	A	0.1376
**CXCR4E**	T	T	T	G	0.0482
**BLANK**					0.0611

The *CXCR4*_A haplotype (either homozygous or heterozygous) was associated with a higher mobilized CD34+ cell count, in comparison with other haplotypes (63.7 [4–237.6] *vs* 54.35 [6.5–196.3], p = .03) (Fig 1C and Table 12). We did not find any other significant association between haplotypes for other genes and CD34+ cells mobilization (Table 10).

**Table 12:** Impact of *VCAM/VLA4* and *SDF1/CXCR4* haplotypes on CD34+ cell mobilization.

haplotypes								haplotypes		
*SDF1_A*	AA	AB	AC	AD	AE	A/other	non A	*SDF1_A*	non A	A
Number of values	21	30	33	26	25	114	226	Number of values	226	135
Minimum	12,7	7,1	8,9	11,6	12,8	7,1	4	Minimum	4	7,1
25% Percentile	26,05	42,03	27,45	35,6	38,35	35,23	37,03	25% Percentile	37,03	33,9
Median	53,9	66,1	58,5	56,4	61,8	59,2	59,6	Median	59,6	58,5
75% Percentile	88,55	102	99,15	67,88	95	92,63	89,55	75% Percentile	89,55	91,9
Maximum	177,4	193,9	183,3	196,3	186,8	196,3	237,6	Maximum	237,6	196,3
P (Anova)	0.99							P (Student)	0.94	
*SDF1_B*	BB	BA	BC	BD	BE	B/other	non B	*SDF1_B*	non B	B
Number of values	18	30	24	27	32	113	236	Number of values	236	131
Minimum	6,7	7,1	8,8	14,5	8,7	7,1	4	Minimum	4	6,7
25% Percentile	42,48	42,03	24,98	32,2	38,8	36,4	34,98	25% Percentile	34,98	41,2
Median	80,05	66,1	58,4	68,4	54,6	63,8	57,5	Median	57,5	64,4
75% Percentile	105,9	102	106	106,9	82,13	95,45	88,6	75% Percentile	88,6	99,5
Maximum	161,3	193,9	190,6	162,6	237,6	237,6	196,3	Maximum	196,3	237,6
P (Anova)	0.91							P (Student)	0.23	
*SDF1_C*	CC or C-	CA	CB	CD	CE	C/other	non C	*SDF1_C*	non C	C
Number of values	19	33	24	27	23	107	235	Number of values	235	126
Minimum	8,9	8,9	8,8	6,5	19,9	6,5	4	Minimum	4	6,5
25% Percentile	30,6	27,45	24,98	42,3	39,2	36,3	35,7	25% Percentile	35,7	34,38
Median	49,4	58,5	58,4	61	59,2	59,2	59,6	Median	59,6	58,85
75% Percentile	77,5	99,15	106	90,9	90,4	93	89,4	75% Percentile	89,4	91,28
Maximum	119,3	183,3	190,6	183,9	160,8	190,6	237,6	Maximum	237,6	190,6
P	0,98							P (Student)	0.92	
*SDF1_D*	DD or D-	DA	DB	DC	DE	D/other	non D	*SDF1_D*	non D	D
Number of values	14	26	27	27	28	108	245	Number of values	245	122
Minimum	28,9	11,6	14,5	6,5	6,8	6,5	4	Minimum	4	6,5
25% Percentile	34,78	35,6	32,2	42,3	30,65	36,35	35,1	25% Percentile	35,1	35,6
Median	47,9	56,4	68,4	61	55,85	59,5	59,2	Median	59,2	59,25
75% Percentile	84,73	67,88	106,9	90,9	89,85	89,85	90,25	75% Percentile	90,25	89,55
Maximum	183,7	196,3	162,6	183,9	175,4	196,3	237,6	Maximum	237,6	196,3
P	0,99							P (Student)	0.91	
*SDF1_E*	EE or E-	EA	EB	EC	ED	E/other	non E	*SDF1_E*	non E	E
Number of values	14	25	32	23	28	108	239	Number of values	239	122
Minimum	4	12,8	8,7	19,9	6,8	6,8	6,5	Minimum	6,5	4
25% Percentile	38,83	38,35	38,8	39,2	30,65	37,35	34,9	25% Percentile	34,9	37,65
Median	57,7	61,8	54,6	59,2	55,85	59,4	59,6	Median	59,6	59,15
75% Percentile	67	95	82,13	90,4	89,85	88,48	92,4	75% Percentile	92,4	84,18
Maximum	107,5	186,8	237,6	160,8	175,4	237,6	196,3	Maximum	196,3	237,6
P	0.96							P (Student)	0.78	
**haplotypes**										
*CXCR4_A*	AA	AB	AC	AD	AE	A/other	non A	*CXCR4_A*	non A	A
Number of values	35	80	40	38	7	205	158	Number of values	158	189
Minimum	4	7,4	8,9	11	22,6	7,4	6,5	Minimum	6,5	4
25% Percentile	34	34,1	37,95	38,18	44,5	36,4	36,83	25% Percentile	36,83	37,4
Median	64,8	60,1	63,35	68,1	57,2	63	54,35	Median	54,35	63,7
75% Percentile	115,5	96,78	89,93	109,5	83,4	92,7	84,35	75% Percentile	84,35	98,95
Maximum	190,6	237,6	186,8	183,9	175,8	237,6	196,3	Maximum	196,3	237,6
P	0.37							P (Student)	0,03	
*CXCR4_B*	BB	BA	BC	BD	BE	B/other	non B	*CXCR4_B*	non B	B
Number of values	35	80	31	31	13	155	168	Number of values	168	190
Minimum	17	7,4	6,5	6,8	7,1	6,5	4	Minimum	4	6,5
25% Percentile	42,5	34,1	35,7	25,9	22,3	32,2	41,38	25% Percentile	41,38	33,03
Median	58,2	60,1	59,6	47,3	63	58,8	60,6	Median	60,6	58,5
75% Percentile	95,6	96,78	80,4	90,9	93,6	90,9	90,08	75% Percentile	90,08	91,68
Maximum	196,3	237,6	168,5	147,9	119,3	237,6	190,6	Maximum	190,6	237,6
P	0.65							P (Student)	0.31	
*CXCR4_C*	CC or C-	CA	CB	CD	CE	C/other	non C	*CXCR4_C*	non C	C
Number of values	9	40	31	16	9	94	262	Number of values	262	103
Minimum	32,2	8,9	6,5	21,7	41,9	6,5	4	Minimum	4	6,5
25% Percentile	48,3	37,95	35,7	31,93	43,55	40,58	34,98	25% Percentile	34,98	41,63
Median	60,6	63,35	59,6	47,5	54,2	59,3	59,6	Median	59,6	59,5
75% Percentile	79,13	89,93	80,4	62,1	89,1	83,8	92,63	75% Percentile	92,63	82,6
Maximum	120,9	186,8	168,5	105,4	111	186,8	237,6	Maximum	237,6	186,8
P	0.67							P (Student)	0.29	
*CXCR4_D*	DD or D-	DA	DB	DC	DE	D/other	non D	*CXCR4_D*	non D	D
Number of values	7	29	31	16	4	80	271	Number of values	271	
Minimum	32,8	11	6,8	21,7	38	6,8	4	Minimum	4	6,8
25% Percentile	56	40,85	25,9	31,93	40,98	35,55	35	25% Percentile	35	36,3
Median	79,9	76,6	47,3	47,5	50,65	52,6	59,6	Median	59,6	56
75% Percentile	85,7	109,7	90,9	62,1	63,18	91,73	90	75% Percentile	90	90,9
Maximum	162	183,7	147,9	105,4	67,1	183,7	237,6	Maximum	237,6	183,7
P	0.31							P (Student)	0.55	
*CXCR4_E*	EE or E-	EA	EB	EC	ED	E/other	non E	*CXCR4_E*	non E	E
Number of values	2	7	13	9	4	33	323	Number of values	323	
Minimum	20	22,6	7,1	41,9	38	7,1	4	Minimum	4	7,1
25% Percentile	20	44,5	22,3	43,55	40,98	42,5	35,3	25% Percentile	35,3	41,9
Median	39	57,2	63	54,2	50,65	54,2	60,1	Median	60,1	54,2
75% Percentile	58	83,4	93,6	89,1	63,18	85,8	92	75% Percentile	92	83,4
Maximum	58	175,8	119,3	111	67,1	175,8	237,6	Maximum	237,6	175,8
P	0.80							P (Student)	0.31	

### Clinical versus biological factors in predicting CD34+ cells mobilization

**Fig 2:**
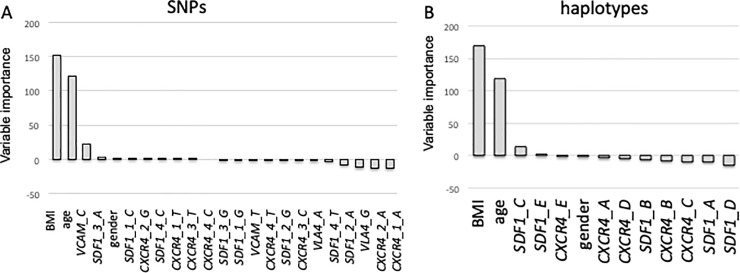
Regression analyses evaluating importance of clinical and molecular variables (A: SNPs and B: haplotype for the 4 studied genes) in predicting CD34+ cells mobilization.

Two random forests for linear regression model were built in order to study importance of clinical and molecular variables on CD34+ cell mobilization [20, 21]. As shown in Fig 2, clinical factors were strongly associated with CD34+ cell mobilization contrary to molecular data, either taking SNP or haplotypes into account. Thus, we concluded that age and BMI data alone are sufficient to predict CD34+ cell mobilization in the context of ASCT.

## Discussion

ASCT is used for the treatment of patients with a variety of severe malignant or non-malignant hematological disorders, either constitutional or acquired. A main challenge in ASCT is to rapidly identify and select a suitable donor, from whom to collect sufficient numbers of hematopoietic cells and progenitors; when apheresis is used to collect allogeneic peripheral blood stem cells, most transplant programs have defined minimal number of CD34+ cells to procure as a mean to ensure rapid engraftment and establish hematopoietic chimerism in the recipient. The extent of CD34+ cell mobilization varies significantly among donors, mainly depending on age, gender or weight, possibly also on genetic variations. [11–13] However, few published studies conducted on CD34+ cell mobilization included a multivariate analysis simultaneously considering biological and genetic variables. Here, we analyzed biological and genetic parameters described to influence CD34+ cell mobilization after G-CSF administration in 367 consecutive volunteer healthy donors that were homogeneously cared for at a single institution. Our results suggest that age, BMI, and possibly sex mostly influence the response to G-CSF.

Larger registry studies conducted in unrelated–and thus on younger—donors already identified the influence of these factors on stem cell mobilization; our single-institution cohort of related donors offers the advantage of harmonized mobilization and collection procedures. In addition, published studies on unrelated donors do not include the analysis of genetic variants. In our cohort, genetic variations in genes whose products are known to play an important role in stem cell egress out of the bone marrow little affects the results of mobilization and collection procedures used in the clinical context of ASCT. Such associations were evidenced in a much smaller cohort of 112 donors in a previously published report [11]; donors in this study were however younger than in our cohort (38 years old vs 50 years old), which may affect the results of such studies. While the number of analyzed individuals may appear small, the cohort was large enough to allow for the confirmation of the predictive value of age and BMI, variables whose predictive value for mobilization was already demonstrated in other contexts. The random forest analysis that was performed suggests that addition of genetic factors will not add to the predictive value of the model, and that increasing the size of the cohort is unlikely to change our conclusions.

Given these uncertainties, it is unlikely that screening donors for these individual SNPs could produce relevant information to guide clinical practice. In addition, we further analyzed whether haplotypes could be associated with different levels of response to rhG-CSF, but failed to evidence such a relation. Nevertheless, we found that the *VCAM1*-rs1041163 CC homozygous variant is associated with a lower mobilization, similarly to what was previously described in a 112 healthy individual cohort in a study by Martin-Antonio et al; [11] the authors also found that this variant was associated with a lower PB CD34+ cells mobilization after G-CSF treatment. By contrast, in a recent study on a smaller cohort of 46 patients, the frequency of this VCAM1 CC allele was higher in the good mobilizer group. [13] Our study also confirms previous negative findings on the influence of the *SDF1*-rs1801157 polymorphism in two other cohort of 463 and 515 donors on CD34+ cell mobilization. [15, 22]

CD44, another gene that encodes a molecule involved in adhesive and chemotactic interactions of CD34+ cells within the bone marrow niche [14], or *DGKB*, a crucial regulator of glycerolipid metabolism [13] are also involved in stem cell retention and mobilization, and deserve further exploration.

In conclusion, our study provides additional evidences supporting a relation between clinical donor characteristics such as BMI, age, and possibly sex and the biological response to rhG-CSF used as a CD34+ cell mobilization agent in view of cell procurement for ASCT. Together with previously published work, it does not support a strong relationship between genetic polymorphisms in the sequence coding for functionally important molecules–and pharmacological targets–involved in hematopoietic progenitor cell trafficking. Since interactions of stem cells with the bone marrow niches involve multiple molecular actors, it is possible that a more comprehensive exploration such as GWAS could identify genetic patterns associated with more or less profound response to rhG-CSF. Existing evidences however do not support donor explorations for clinical applications. To ensure rapid hematopoietic recovery after HSCT, optimization of CD34+ cell collection during apheresis mostly relies on tailoring procedural parameters to donor characteristics, including immediate pre-apheresis measurement of CD34+ cell numbers in the peripheral blood.

## Supporting information

S1 File Table A. SDF1 haplotypes and their frequencies estimated by Gene[rate] based on rs1801157, rs266087, rs2297630, rs266085, rs1413519 observed polymorphisms.
Table B. CX4CR1 haplotypes and their frequencies estimated by Gene[rate] based on rs16832740, rs2228014, rs2680880 and rs12691874observed polymorphisms.
Table C. Main SDF1 haplotypes frequencies estimated with an *a priori* by Gene[rate].
Table D. Main CXCR4 haplotypes frequencies estimated with an *a priori* by Gene[rate].
(DOCX)
