# Supplementary material for: Analysis of a large single institution cohort of related donors fails to detect a relation between SDF1/CXCR4 or VCAM/VLA4 genetic polymorphisms and the level of hematopoietic progenitor cell mobilization in response to G-CSF
Source: PLoS One. 2020 Mar 5;15(3):e0228878. doi: 10.1371/journal.pone.0228878 (PMC7058310; doi:10.1371/journal.pone.0228878)
Supplement: S1 File — Table A. SDF1 haplotypes and their frequencies estimated by Gene[rate] based on rs1801157, rs266087, rs2297630, rs266085, rs1413519 observed polymorphisms. Table B. CX4CR1 haplotypes and their frequencies estimated by Gene[rate] based on rs16832740, rs2228014, rs2680880 and rs12691874observed polymorphisms. Table C. Main SDF1 haplotypes frequencies estimated with an a priori by Gene[rate]. Table D. Main CXCR4 haplotypes frequencies estimated with an a priori by Gene[rate]. (DOCX) [file pone.0228878.s001.docx]

Genotyping and haplotype estimation

SNPs were typed either by Snap Shot extension primer or by direct sequencing (SBT). Snap Shot extension primer data were analyzed using GeneMapper v4.0 with specific detection parameters and sequencing data were analyzed with Codon code Aligner program. Haplotypes frequencies based on SNPs on a same gene, i.e. SDF1 and CX4CR1, were estimated by Gene[rate] computer tool package (Tables A and B). Given the low number of estimated haplotypes (9 haplotypes for SDF1 with 5 haplotypes representing a cumulated frequency of 99.3% and 8 haplotypes for CX4CR1 with 5 haplotypes representing a cumulated frequency of 98.9%), 5 haplotypes were encoded for each gene with an a priori and data were reanalyzed according to this new nomenclature. Using an in-house computer program, data output files (.txt) were formatted into files readable by the “*Phenotype*” application of the Gene[rate] computer tool package (21) (Table C and D). *Phenotype* uses two input files (i.e. a kit description file and a reaction data file) to determine the phenotype. With this new coding, blank haplotype, accounting for samples that genotype profiles do not match with these expected haplotypes, represented respectively 5.9% and 4.9% for SDF1 and CXCR4.

Table A. SDF1 haplotypes and their frequencies estimated by Gene[rate] based on rs1801157, rs266087, rs2297630, rs266085, rs1413519 observed polymorphisms

| Haplotype | Fq |
| --- | --- |
| A~A~G~T~G | 0,2178 |
| G~A~G~T~G | 0,2022 |
| G~G~A~C~G | 0,2005 |
| G~G~G~C~G | 0,1872 |
| G~G~G~C~C | 0,1851 |
| G~G~G~T~G | 0,0021 |
| A~G~G~T~G | 0,002 |
| G~A~G~T~C | 0,0017 |
| G~A~G~C~G | 0,0014 |

Table B. CX4CR1 haplotypes and their frequencies estimated by Gene[rate] based on rs16832740, rs2228014, rs2680880 and rs12691874observed polymorphisms

| Haplotype | Fq |
| --- | --- |
| T~C~T~G | 0,322 |
| T~C~T~A | 0,3151 |
| C~C~A~G | 0,1618 |
| T~C~A~A | 0,1394 |
| T~T~T~G | 0,0506 |
| C~C~T~G | 0,0048 |
| T~C~A~G | 0,0044 |
| C~C~A~A | 0,0019 |

Table C. Main SDF1 haplotypes frequencies estimated with an *a priori* by Gene[rate]

| Haplotype | rs1801157 | rs266087 | rs2297630 | rs266085 | rs1413519 | Fq |
| --- | --- | --- | --- | --- | --- | --- |
| *SDF1A* | A | A | G | T | G | 0,2037 |
| *SDF1B* | G | A | G | T | G | 0,1925 |
| *SDF1C* | G | G | A | C | G | 0,1906 |
| *SDF1D* | G | G | G | C | G | 0,1778 |
| *SDF1E* | G | G | G | C | C | 0,1763 |
| blank |  |  |  |  |  | 0,0591 |

Table D. Main CXCR4 haplotypes frequencies estimated with an *a priori* by Gene[rate]

| Haplotype | rs16832740 | rs2228014 | rs2680880 | rs12691874 | FQ |
| --- | --- | --- | --- | --- | --- |
| CXCR4A | T | C | T | G | 0,3053 |
| CXCR4B | T | C | T | A | 0,301 |
| CXCR4C | C | C | A | G | 0,1575 |
| CXCR4D | T | C | A | A | 0,1369 |
| CXCR4E | T | T | T | G | 0,0496 |
| blank |  |  |  |  | 0,0496 |

The assay technique presented here has two other major advantages, i.e., computer-assisted data interpretation and primer adaptability. By allowing use of a home-made formatting program and Gene[Rate] program tools (21) to handle files exported from Genemapper 4.0, the present technique not only permits simultaneous consideration of all SNPs in a single run of the program but also lowers the risk of human error during processing. The assay is adaptable since new PCR and/or extension primers can be added for analysis of new polymorphisms. However, primer extension technology presents two potential limitations; neither it is possible to detect new alleles nor to determine the haplotype phase of the SNPs detected.

21. Nunes J. Generate: tools for analysis and handling of data with ambiguities. Laboratory of Anthropology, Genetics and Peopling History, University of Geneva, Switzerland, 2006. <http://geneva.unige.ch/generate/>

Statistical analysis

Statistical analysis was performed using SPSS software (SPSS 19.0 for Windows; SPSS Inc., Chicago, IL). The primary endpoint was influence of SNPs on CD34-positive cell count/mL peripheral blood on day 5 of G-CSF treatment. For continuous variables, median and extreme values are presented. Differences of medians in univariate analyses have been analyzed with the t-test for independent samples. T-test were considered as significant when two-tailed p-values were <.05, except for clinical or molecular factors that has already been associated with a modification with mobilization in previous studies (age, gender, BMI and VCAM1_rs1041163 CC homozygous variant). In these case, given the expected influence on mobilization, we used a one-tailed p-value <.05.
